# Impacts from Wildfires on Livestock Health and Production: Producer Perspectives

**DOI:** 10.3390/ani11113230

**Published:** 2021-11-12

**Authors:** Kathleen C. O’Hara, Juliana Ranches, Leslie M. Roche, Tracy Kay Schohr, Roselle C. Busch, Gabriele U. Maier

**Affiliations:** 1 Center for Animal Disease Modeling and Surveillance (CADMS), School of Veterinary Medicine, University of California, Davis, CA 95616, USA; kcohara@ucdavis.edu; 2 Eastern Oregon Agricultural Research Center (EOARC), Oregon State University, Burns, OR 97720, USA; juliana.ranches@oregonstate.edu; 3 Department of Plant Sciences, University of California Davis, Davis, CA 95616, USA; lmroche@ucdavis.edu; 4 University of California Cooperative Extension, Plumas-Sierra-Butte Counties, Quincy, CA 96130, USA; tkschohr@ucanr.edu; 5 Department of Population Health & Reproduction, School of Veterinary Medicine, University of California Davis, Davis, CA 95616, USA; rcbusch@ucdavis.edu

**Keywords:** wildfires, livestock, cattle, production losses, smoke

## Abstract

**Simple Summary:**

Wildfires are increasing in frequency and severity across the Western United States. Efforts to understand the health impacts on humans are widespread and expanding; however, very little is known about the impact of wildfires and smoke exposure on livestock. This work presents the results of a survey of cattle, sheep, and goat producers in California, Oregon, and Nevada, on their experiences during the 2020 wildfire season. While few direct impacts of fires were reported among the 70 responses, 26% of respondents reported they had to evacuate livestock and 19% reported pasture losses. Indirect losses from smoke exposure, including pneumonia and reproductive losses were reported more broadly. This preliminary work highlights the need to better understand impacts of wildfires on livestock and how policy changes can help support the livestock production industry through these crises.

**Abstract:**

Wildfires are increasing in frequency and severity across the Western United States. However, there is limited information available on the impacts these fires are having on the livelihood of livestock producers and their animals. This work presents the results of a survey evaluating the direct and indirect impacts of the 2020 wildfire season on beef cattle, dairy cattle, sheep, and goat, producers in California, Oregon, and Nevada. Seventy completed surveys were collected between May and July 2021. While dairy producers reported no direct impacts from the fires, beef, sheep, and goat producers were impacted by evacuations and pasture lost to fires. Only beef producers reported losses due to burns and burn-associated deaths or euthanasia. Dairy, beef, sheep, and goat producers observed reduced conception, poor weight gain, and drops in milk production. All but dairy producers also observed pneumonia. Lower birthweights, increased abortion rates, and unexplained deaths were reported in beef cattle, sheep, and goats. This work documents the wide-ranging impacts of wildfires on livestock producers and highlights the need for additional work defining the health impacts of fire and smoke exposure in livestock, as well as the policy changes needed to support producers experiencing direct and indirect losses.

## 1. Introduction

Globally, climate change has been predicted to increase the risk of wildfires in most areas of the world [1]. In 2020, more than 59,000 wildfires burned 4.09 million hectares (ha) in the United States, with more than 90% of this hectarage in the West [2]. The frequency and intensity of wildfires has continued to increase annually across the Western United States [3,4]. The National Interagency Fire Center (NIFC) reported 10,431 fires burning 1,656,034.6 ha in California, 2215 fires burning 461,994.2 ha in Oregon, and 770 fires burning 104,924.9 ha in Nevada, during 2020 alone [5]. Five of the top 20 largest and most destructive wildfires in California history occurred in 2020 [6,7]. Ongoing drought conditions, and drier climatic conditions, are predicted to contribute to intensifying wildfires [8,9].

Across both human and animal research, additional data is needed on the effects of natural exposure to wildfire smoke. With the increased occurrence of wildfires, literature studying the impacts of wildfires on human health has been rapidly expanding [10,11,12,13,14,15,16,17,18,19]. The evaluation of smoke and particulate matter exposure and human health outcomes has been a particular focus [20,21,22,23,24]. Literature on wildfire and smoke impacts in livestock on the other hand is very limited. 

The predominance of available literature on smoke inhalation in animals is based on experimental studies. Ovine models in particular have been used for decades to study the pathophysiology of smoke inhalation in humans [25,26,27,28,29,30,31,32,33,34,35,36], and to evaluate potential treatment options [37,38,39,40,41,42,43,44,45,46]. Rodent models of smoke inhalation have been used toward the same research goals, though the inferences to livestock physiology are more limited [47,48,49,50,51]. While these data provide some initial insights into the pathophysiology of smoke exposure in livestock, the lack of corresponding data on natural exposures limits the ability to make evidence-based inferences about the impacts of natural wildfire smoke exposure on animal production and health outcomes. 

A few recent publications have attempted to summarize what is known, and presented some new findings, about animals naturally exposed to wildfire smoke. A review out of Australia summarized the literature on brushfire smoke exposure in cattle [52]. The article highlighted that the available literature is focused on the impact of particulate matter emissions, primarily in humans. Some studies were available on ambient air pollution associated with feedlots, however, little to no data about smoke associated with fires was reported. Few to no health impacts on cattle were reported in the review, however, there were no studies available directly evaluating the impacts of smoke exposure in these animals [52]. Researchers at the University of California, Davis, recently published a study on cats hospitalized with burns and smoke inhalation following the 2017–2018 California wildfires; the cats were shown to have significant cardiovascular changes based on serial echocardiograms [53]. Rhesus macaques housed at the California National Primate Research Center and naturally exposed to wildfire smoke were reported to have pregnancy losses following heavy smoke exposure [54]. Additionally, early data suggests that immune suppression may be evident in macaques even 12 years following smoke exposure [54]. These latest studies both document an association between smoke exposure and diverse pathologies, and highlight the lack of peer-reviewed research in this area, underscoring the need for additional information.

While peer-reviewed articles are limited, agricultural support organizations, cooperative extension, universities, government, and news media have produced and shared numerous guidance documents on protecting livestock, ranches, range, and pasturelands from wildfires [55,56,57,58,59,60,61,62,63,64]. Additionally, guidance on the treatment of burned animals and the care of animals following exposure to fires is available [57,65,66,67,68,69]. While some losses, such as that of feed and pastureland, have been well documented, there is less information about livestock production losses [70,71]. Current welfare and indemnity programs are focused on documented and easily quantifiable direct losses, suggesting a gap in policy support for wildfire associated livestock production losses [72].

This work aimed to collect information from beef cattle, dairy cattle, sheep, and goat producers in California, Oregon, and Nevada, on their experiences and associated losses during the 2020 wildfire season, to better understand potential direct and indirect losses producers may be observing associated with wildfires.

## 2. Materials and Methods

A semi-structured questionnaire, including a combination of multiple choice and open-ended questions, was developed using Qualtrics software, version May 2021 [73]. Questions assessed the direct and indirect impacts of wildfires on livestock following the 2020 wildfire season (Supplementary File S1). Direct impacts included losses associated with burns and burn-associated deaths and euthanasia, as well as evacuations and pasture losses. Indirect impacts were those primarily associated with smoke inhalation or the stressors of movements, evacuation, or confinement following the primary fire threat. Survey results represent a convenience sampling of observational data [74]. The questionnaire was distributed to contacts and listservs associated with livestock producers in California, Oregon, and Nevada, including University of California Agriculture and Natural Resources, the California Cattlemen’s Association, California Wool Growers Association, Western United Dairies, Oregon State University Extension Service, and the Oregon Cattlemen’s Association. The survey was opened and electronically distributed 18 May 2021 and was closed 31 July 2021. Using the Qualtrics platform, producers were able to respond using smartphones or computers. Producers were encouraged to participate in the survey even if they did not suffer losses associated with wildfires.

Data were cleaned and analyzed in R Studio [75]. Previews (instances when the survey was opened and trialed during the development phase) and incomplete submissions were discarded. Seventy completed responses qualified for analysis using these criteria. Responses were divided based on the type of livestock owned. Proportion of responses were calculated as the number of respondents who selected a given answer divided by the number of responding producers for that given livestock type. Some producers owned multiple livestock types and were thus included in the denominators for each respective type.

## 3. Results

**Figure 1:**
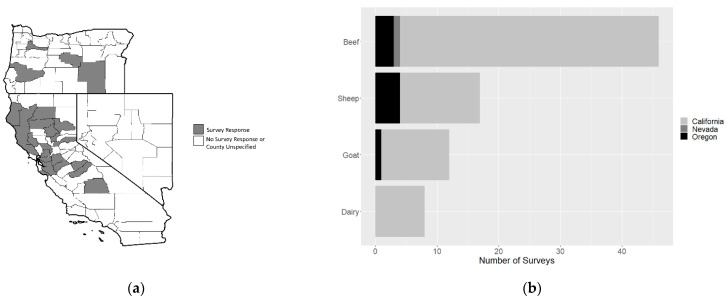

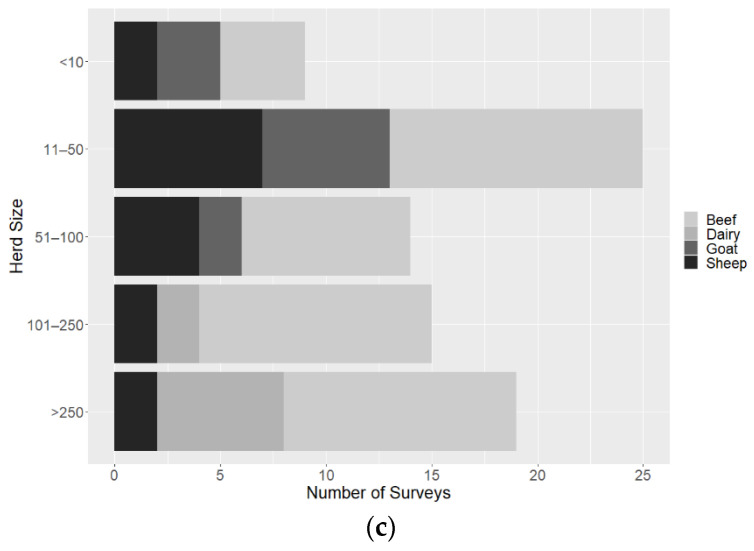
Livestock producers responding to a survey on 2020 wildfire impacts on livestock production and health, (**a**) mapped by county of origin (note: 25/70 respondents did not identify their county), (**b**) by livestock type and state, (**c**) by livestock type and herd size.

A total of 70 completed responses were analyzed, 61 from California, 8 from Oregon, and 1 from Nevada. The counties from which responses were received were mapped (Figure 1a; 25/70 respondents did not provide county information) and the distribution of responses by livestock type and state were plotted (Figure 1b). Among respondents, 8 owned dairy cattle, 46 owned beef cattle, 17 owned sheep, and 11 owned goats. Eleven respondents owned multiple species. Survey respondents also reported their herd sizes (Figure 1c). All dairy respondents had more than 100 cattle, while goat respondents had fewer than 100. The survey captured beef and sheep producers with herd sizes ranging from less than 10 to greater than 250. 

**Figure 2:**
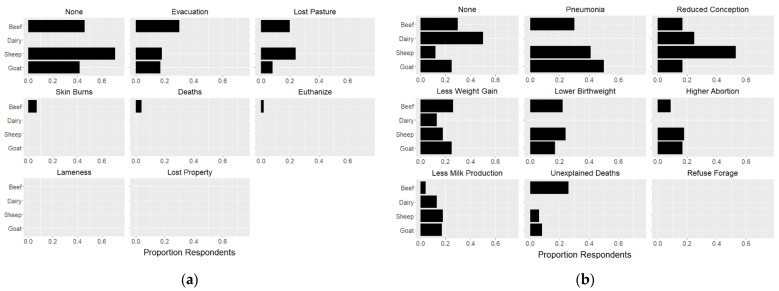
Proportion of survey respondents reporting they experienced or observed (**a**) direct and/or (**b**) indirect effects of 2020 wildfires by livestock type.

The proportion of respondents, by livestock type, reporting direct and/or indirect losses was calculated (Figure 2). Responding dairy producers reported no direct effects. Beef cattle, sheep, and goat producers reported being impacted by evacuations (30%, 18%, and 17%, respectively) and lost pasture (20%, 24%, and 8%, respectively); only beef cattle producers reported direct losses due to skin burns (7%), euthanasia following burns (2%), or direct deaths (4%). Indirect impacts, those presumably attributed to smoke inhalation or evacuation and movement stressors, were more widely reported. Pneumonia was reported by beef (30%), sheep (41%), and goat (50%) producers, though not by dairy producers. Reduced rates of conception were reported across livestock types, with 50% of sheep producers reporting drops in conception. Negative impacts on weight gain were also reported across all livestock types, impacting 13–26% of producers. Additional reproductive losses, including lower birthweights and higher rates of abortion were reported among beef cattle, sheep, and goat producers, with sheep reporting such losses most commonly. Decreased milk production was reported across livestock types, with 13% of dairy producers reporting losses. Unexplained deaths were reported by 26% of beef cattle producers, and to a lesser extent among sheep (6%) and goat (8%) herds. No producers reported refusal to eat forage or graze pasture due to ash or smoke.

In addition to the direct and indirect losses reported in Figure 2, open-ended questions about losses provided some additional insights. Six beef producers, one sheep producer, and one goat producer reported pneumonia or respiratory issues due to heavy smoke. Erratic behavior resulting in broken fences was noted by a dairy producer. Two beef and one sheep producer mentioned deaths or abortions in the immediate days after heavy smoke exposure. One beef and one goat producer mentioned the stress of evacuations, with one beef producer also reporting loss of dam–calf pairings in the chaos. Disruption of conception cycles was noted by one beef and one sheep producer. One beef cattle producer in Oregon who sheltered in place used all of their winter hay while keeping the animals corralled during the fire. The negative impact of ash and fire debris on water quality was also a concern expressed by a beef producer.

## 4. Discussion

This preliminary study is one of the first to report producers’ perspectives on the direct and indirect impacts of wildfires on livestock in the Western United States, providing critical insights from an understudied at-risk population. Results show beef, sheep, and goat producers appear to be more impacted by evacuations and loss of pasture, than other direct losses such as livestock burns and deaths. The most commonly reported indirect impact was pneumonia secondary to smoke exposure. Bovine respiratory disease has been linked to stress and immune suppression, as well as congregation of cattle or lack of biosecurity [76,77]. The stress of evacuation or movement, in combination with congregation and possible commingling with animals from other herds and handling by outsiders, may have been a contributing factor in the respiratory disease observed by survey participants. Interestingly, dairy producers responding to the survey failed to observe clinical respiratory disease but reported a drop in milk production as well as decreased fertility. One explanation could be that the impact on dairy cattle was less traumatic and did not include an increase in congregation, handling, or movement, as none of the dairy producers reported evacuation. Producers that had to evacuate were also likely closer to a wildfire and thus may have also been exposed to higher concentrations of particulate matter. Furthermore, changes in milk production can easily be measured on dairies and are of utmost importance to their business, therefore, they are also more likely to be noted by dairy producers. The aforementioned studies on ovine models of smoke inhalation leave little doubt that smoke inhalation in ruminants may contribute to lung pathology, compounding the effect of stress and associated immune suppression. Overall, the available evidence across species appears to be consistent. 

Impacts on reproduction, including reduced conception, low birthweight, poor weight gain, and even abortion, were reported across species, with participating sheep producers representing the largest proportion of herds impacted. This finding is particularly interesting given the lack of previous literature on this topic, and the documentation of reduced birthweight and increased preterm births in women exposed to smoke [16]. Disruption of cyclicity may have been partially attributable to stress related to nearby wildfires. Stunted offspring observed by survey respondents may be due to the same pathophysiological processes that are suspected to contribute to premature births in humans [16]. These may include the cytotoxic effects of inhalation of oxygen-free radicals [78], or the disruption of fetal–maternal circulation [79], which have been shown to result in low birth weights and other poor birth outcomes [80]. A recent study reported miscarriage in Rhesus macaques naturally exposed to wildfire smoke, further supporting the need to expand research of these reproductive impacts of smoke on animals [54].

Losses such as the ones reported by survey respondents are likely to have a large impact on the livelihood of producers, and further investigation is warranted. Across direct and indirect impacts, those animals that are most reliant on pasture, and thus more highly exposed, were the most affected. More detailed analysis of the impact of wildfires on forage and water quality post-fire, which may explain some of these observations, are ongoing.

As a convenience sampling, the results of this study are not representative of all producers, livestock species, or areas impacted by wildfires. Evaluating the response numbers against the total number of producers in the participating states for the different livestock commodities shows that only a small proportion filled out surveys. For example, according to the 2017 USDA census of agriculture, there were 4587 beef farms, 1653 dairy farms, 3807 sheep and lamb flocks, and 3938 goat herds in California [81]. However, the purpose of the study was not to estimate, with a high degree of confidence, the percent of livestock impacted, but to assess whether there is any evidence to suggest that smoke exposure due to wildfires may pose health risks to livestock. In addition, these outreach efforts were likely unable to reach the entire population of livestock producers, especially not those with limited or no online access. Rather, this study focused on those producers who are part of local livestock associations or known to Cooperative Extension advisors. Given the preliminary nature of the survey, where the main goal was hypothesis generation, statistical techniques to adjust responses to better reflect the sampled population, such as survey-weighting, were not attempted. As such, results should not be interpreted as providing estimates of affected producers in each of the targeted commodities or states. Results do, however, provide initial insights to direct future, more detailed, work. Additionally, the authors acknowledge that those producers who were impacted by wildfires may be more likely to complete surveys on this topic, which may bias results toward an overestimation of the risks. However, additional work is needed to determine if such bias occurred. 

As climate change continues to drive weather extremes, the Western U.S. can expect to continue to see prolonged drought conditions and the ongoing increase in the number and severity of wildfires. This work begins to document the indirect impact of these wildfires on livestock producers. While the impacts of wildfires, and particularly smoke, are being broadly investigated in humans, very little information is available on livestock. Difficulty in estimating effects of smoke and other sequelae of wildfires on livestock health stem from the fact that data on livestock health and production parameters in privately owned herds is not readily available. Additionally, factors such as feed availability during drought years may confound results. While limited in scope, this work highlights the reality of losses and impacts on livestock, and the need for additional work in this area. Many of the losses reported, including having to evacuate animals, are not covered by existing relief programs. Direct losses not resulting in death are not eligible for indemnity [82]. While some of the effects of wildfires are difficult to mitigate for pasture-raised animals, it is critical that we understand the impact on livestock producers and shape policy to support this vital industry. This study supports the need for additional investigations into the health impacts of wildfires and smoke on livestock, and the need to better quantify the prevalence and scale of effects on respiratory and reproductive health in particular. Further knowledge in this area may help with the development of best practices, such as the use of metaphylaxis or other interventions, to mitigate any adverse effects on health and production of grazing livestock. Additionally, strategies to expedite veterinary support and returns to normal reproductive cycles after wildfire exposure are needed. Further knowledge can provide context for extension agents to work with producers to mitigate future livestock production losses due to indirect impacts of wildfire. Finally, as more is known about the extent and areas in which livestock production losses are occurring, assessments of current relief programs should be made to determine if producers are eligible for, and receiving, the aid they need. 

## 5. Conclusions

This study provides one of the first reports of direct and indirect losses livestock producers are experiencing due to wildfires in the Western United States. While this work represents a convenience sampling of a small number of producers, the results support the need for ongoing research and funding in this area to better quantify losses for targeted mitigations and policy-backed aid and indemnity. The extensive reproduction-associated losses in particular should be highlighted for additional study.

## Data Availability

The complete dataset is available upon request. Please contact Gabriele Maier, gumaier@ucdavis.edu.

## Supplementary Materials

The following are available online at https://www.mdpi.com/article/10.3390/ani11113230/s1, Supplementary File S1. Qualtrics survey administered to livestock producers from California, Oregon, and Nevada between May and July 2021 to understand the direct and indirect impacts of the 2020 wildfire season on livestock health and production.
