# Supplementary material for: Impacts from Wildfires on Livestock Health and Production: Producer Perspectives
Source: Animals (Basel). 2021 Nov 12;11(11):3230. doi: 10.3390/ani11113230 (PMC8614491; doi:10.3390/ani11113230)
Supplement: Supplementary file 1 [file animals-11-03230-s001.zip › animals-1428520-supplementary.pdf]

Default Question Block

.

University of California in collaboration with **Oregon State University** developed this survey to gauge the impact of the 2020 wildfires on grazing livestock health and production.

The researchers at UC Davis involved in this survey are part of UC Davis Agriculture and Natural Resources / UC Davis Veterinary Medicine Extension. The researcher at OSU is part of the Eastern Oregon Agricultural and Research Center, College of Agricultural Sciences, and Extension service.

Our programs focus on finding science-based solutions in animal health and livestock management. We work with livestock producers and land managers of all sizes to

address concerns and find innovative solutions that support livestock production.

The objective of this survey is to gather information to better understand to what extent cattle, sheep, and goats have been impacted by direct exposure to wildfires or by indirect exposure to smoke inhalation during the fire season. Even if you do not think your livestock were impacted by wildfires in 2020, we would also like to hear from you.

All questions are related to the 2020 wildfire season between May and December 2020.

The survey should take about 3 - 5 minutes to complete. Your participation in this survey is completely voluntary, and your responses will be kept anonymous.

If you have **any questions, please contact Gabriele Maier** (University of California) at gumaier@ucdavis.edu **or Juliana Ranches** (Oregon State University) at juliana.ranches@oregonstate.edu

.

All questions are related to the 2020 wildfire season between May and December 2020.

1.

What type of livestock do you have?

Please select all that apply

- ☐ Dairy cattle
- ☐ Beef cattle
- ☐ Sheep
- ☐ Goats

D-1.

Please share with us how your DAIRY CATTLE were impacted in the 2020 wildfire season between May and December 2020.

D2.

Please indicate your DAIRY Cattle herd size:

- ○ less than 10
- ○ 11-50

- ○ 51-100
- ○ 101-250
- ○ more than 250

D3.

Please select the state and indicate the county or counties (in the text entry box) where DAIRY CATTLE were between May and December 2020:

- ☐ California
- ☐ Oregon
- ☐ Arizona
- ☐ Nevada
- ☐ Other:

D4.

Have your DAIRY CATTLE been directly affected by a wildfire in 2020?

Please select all that apply.

- ☐ No

- ☐ We had livestock with skin burns.
- ☐ We had livestock with severe lameness after walking on burned fields.
- ☐ We had to evacuate livestock due to a fire.
- ☐ We had to euthanize / cull livestock due to burn injuries.
- ☐ We had livestock die in a fire.
- ☐ We lost pasture or range due to a fire.
- ☐ We lost livestock-related property / facilities due to the fire (hay barn, feed storage, milking parlor, etc)
- ☐ ________ Other:

D5.
Have your DAIRY CATTLE been indirectly affected, presumably by smoke inhalation, from wildfires or immediately after?
Please select all that apply.

- ☐ No
- ☐ We had more livestock with pneumonia than usual during the fire season.
- ☐ We had livestock with less weight gain than usual during the fire season.
- ☐ We had livestock with less milk production than usual during the fire season.
- ☐ We had livestock with reduced conception rates than usual during or immediately after the fire season.
- ☐ We had livestock with a higher late term abortion rate than usual during or immediately after the fire season.

- [ ] We had more than usual low birth weights / stunted offspring / poor-doing offspring during or immediately after the fire season.
- [ ] We had more than usual unexplained livestock deaths during the fire season.
- [ ] We had livestock refuse to eat forage or pasture due to ash and/or smoke.
- [ ] Other: ____________

D6.

If you had fire impacts, which fire do you think was affecting your DAIRY CATTLE the most, if known:

- ○ Name of fire: ____________

B-I.

Please share with us how your BEEF CATTLE were impacted in the 2020 wildfire season between May and December 2020.

B2.

Please indicate your BEEF CATTLE herd size:

- less than 10
- 11-50
- 51-100
- 101-250
- more than 250

B3.

Please select the state and indicate the county or counties (in the text entry box) where BEEF CATTLE were between May and December 2020:

- [ ] California
- [ ] Oregon
- [ ] Arizona
- [ ] Nevada
- [ ] Other:

B4.

Have your BEEF CATTLE been directly affected by a wildfire in 2020?

Please select all that apply.

- ☐ No
- ☐ We had livestock with skin burns.
- ☐ We had livestock with severe lameness after walking on burned fields.
- ☐ We had to evacuate livestock due to a fire.
- ☐ We had to euthanize / cull livestock due to burn injuries.
- ☐ We had livestock die in a fire.
- ☐ We lost pasture or range due to a fire.
- ☐ We lost livestock-related property / facilities due to the fire (hay barn, feed storage, milking parlor, etc)
- ☐ Other: ________

B5.

Have your Beef Cattle been indirectly affected, presumably by smoke inhalation, from wildfires or immediately after? Please select all that apply.

- ☐ No
- ☐ We had more livestock with pneumonia than usual during the fire season.
- ☐ We had livestock with less weight gain than usual during the fire season.
- ☐ We had livestock with less milk production than usual during the fire season.
- ☐ We had livestock with reduced conception rates than usual during or immediately after the fire season.
- ☐ We had livestock with a higher late term abortion rate than usual during or immediately after the fire season.

- ☐ We had more than usual low birth weights / stunted offspring / poor-doing offspring during or immediately after the fire season.
- ☐ We had more than usual unexplained livestock deaths during the fire season.
- ☐ We had livestock refuse to eat forage or pasture due to ash and/or smoke.
- ☐ Other: ______

B6.

If you had fire impacts, which fire do you think was affecting your BEEF CATTLE most, if known:

- ○ Name of fire: ______

S1.

Please share with us how your SHEEP were impacted in the 2020 wildfire season between May and December 2020.

S2.

Please indicate the SHEEP herd size:

- less than 10
- 11-50
- 51-100
- 101-250
- more than 250

S3.

Please select the state and indicate the county or counties (in the text entry box) where SHEEP were between May and December 2020:

- [ ] ______ California
- [ ] ______ Oregon
- [ ] ______ Arizona
- [ ] ______ Nevada
- [ ] ______ Other:

S4.

Have your SHEEP been directly affected by a wildfire in 2020?

Please select all that apply.

- ☐ No
- ☐ We had livestock with skin burns.
- ☐ We had livestock with severe lameness after walking on burned fields.
- ☐ We had to evacuate livestock due to a fire.
- ☐ We had to euthanize / cull livestock due to burn injuries.
- ☐ We had livestock die in a fire.
- ☐ We lost pasture or range due to a fire.
- ☐ We lost livestock-related property / facilities due to the fire (hay barn, feed storage, milking parlor, etc)
- ☐ Other: ________

S5.

Have your SHEEP been indirectly affected, presumably by smoke inhalation, from wildfires or immediately after? Please select all that apply.

- ☐ No
- ☐ We had more livestock with pneumonia than usual during the fire season.
- ☐ We had livestock with less weight gain than usual during the fire season.
- ☐ We had livestock with less milk production than usual during the fire season.
- ☐ We had livestock with reduced conception rates than usual during or immediately after the fire season.
- ☐ We had livestock with a higher late term abortion rate than usual during or immediately after the fire season.

- [ ] We had more than usual low birth weights / stunted offspring / poor-doing offspring during or immediately after the fire season.
- [ ] We had more than usual unexplained livestock deaths during the fire season.
- [ ] We had livestock refuse to eat forage or pasture due to ash and/or smoke.
- [ ] Other: __________

S6.

If you had fire impacts, which fire do you think was affecting your SHEEP most, if known:

- ○ Name of fire: __________

Q27.

Please share with us how your GOATS were impacted in the 2020 wildfire season between May and December 2020.

G2.

Please indicate the GOAT herd size:

- less than 10
- 11-50
- 51-100
- 101-250
- more than 250

G3.

Please select the state and indicate the county or counties (in the text entry box) where GOATS were between May and December 2020:

- [ ] California
- [ ] Oregon
- [ ] Arizona
- [ ] Nevada
- [ ] Other:

G4.

Have your GOATS been directly affected by a wildfire in 2020?

Please select all that apply.

- ☐ No
- ☐ We had livestock with skin burns.
- ☐ We had livestock with severe lameness after walking on burned fields.
- ☐ We had to evacuate livestock due to a fire.
- ☐ We had to euthanize / cull livestock due to burn injuries.
- ☐ We had livestock die in a fire.
- ☐ We lost pasture or range due to a fire.
- ☐ We lost livestock-related property / facilities due to the fire (hay barn, feed storage, milking parlor, etc)
- ☐ [ ] Other:

G5.

Have your GOATS been indirectly affected, presumably by smoke inhalation, from wildfires or immediately after? Please select all that apply.

- ☐ No
- ☐ We had more livestock with pneumonia than usual during the fire season.
- ☐ We had livestock with less weight gain than usual during the fire season.
- ☐ We had livestock with less milk production than usual during the fire season.
- ☐ We had livestock with reduced conception rates than usual during or immediately after the fire season.
- ☐ We had livestock with a higher late term abortion rate than usual during or immediately after the fire season.

- [ ] We had more than usual low birth weights / stunted offspring / poor-doing offspring during or immediately after the fire season.
- [ ] We had more than usual unexplained livestock deaths during the fire season.
- [ ] We had livestock refuse to eat forage or pasture due to ash and/or smoke.
- [ ] Other: ____________

G6.

If you had fire impacts, which fire do you think was affecting your GOATS most, if known:

- ○ Name of fire: ____________

Powered by Qualtrics
